# Using the candidacy framework to understand how doctor-patient interactions influence perceived eligibility to seek help for cancer alarm symptoms: a qualitative interview study

**DOI:** 10.1186/s12913-018-3730-5

**Published:** 2018-12-04

**Authors:** Sara Tookey, Cristina Renzi, Jo Waller, Christian von Wagner, Katriina L. Whitaker

**Affiliations:** 1 Department of Behavioural Science and Health, University College London, WC1E 6BT, London, UK; 2 School of Health Sciences, University of Surrey, Surrey, GU2 7XH UK

**Keywords:** Candidacy, Early diagnosis of cancer, Help-seeking behaviour, Primary health care, Qualitative research, Signs and symptoms

## Abstract

**Background:**

‘Candidacy’ is concerned with the way people consider their eligibility for accessing health services. We used the Candidacy Framework to explore how the doctor-patient relationship can influence perceived eligibility to visit their General Practitioner (GP) among people experiencing cancer alarm symptoms.

**Methods:**

We carried out a secondary analysis of qualitative interviews with 29 women and 33 men, aged ≥50 years experiencing cancer alarm symptoms, recruited through primary care. Interviews focused on symptom experience, help-seeking and primary care use. Framework analysis was used to analyse transcripts with a focus on GP-patient interactions.

**Results:**

Perceived (im)permeability of services acted as a barrier to help-seeking, due to limited availability of appointments, time-limited communication and difficulties asserting candidacy. There was also a focal role of communication in building a positive doctor-patient relationship, with some participants describing resisting offers of appointments as a result of previous negative GP adjudication. These factors not only influenced the current consultation but had longer-term consequences for future consultation.

**Conclusions:**

Candidacy provides a valuable theoretical framework to understand the interactional factors of the doctor-patient relationship which influence perceived eligibility to seek help for possible cancer alarm symptoms. We have highlighted areas for targeted interventions to improve patient-centred care and improve earlier diagnosis.

## Background

The Candidacy Framework describes how people assess their eligibility for accessing health services and how they legitimise their interaction and engagement with services [1, 2]. Candidacy has been applied to understand the different stages of a person’s journey to healthcare, incorporating numerous psychosocial factors which may influence decision-making and behaviour [1, 3–5]. The framework outlines seven ‘over-lapping stages’ involved in the process of identifying, negotiating and asserting candidacy (Table 1), which can be applied to understand how a person comes to seek health care and subsequently navigate services [5].

**Table 1:** Adapted description of the stages of the Candidacy Framework [1]

Stages	Description
1. Identification of candidacy	Process in which a person comes to appraise their issue as needing medical help which legitimises them as a candidate for particular health services.
2. Navigation of services	Knowledge of services provided and appraisal of the practicalities involved in making contact with and accessing services. Includes barriers to accessing services such as needing transport, convenience of appointment times and accumulated costs of attending services.
3. Permeability of services	The ease with which a person can use health services. Includes levels of gate-keeping within a service, the complexity of its referral processes, and the ‘cultural alignment’ of services with the person’s needs and values.
4. Appearance at services	The person’s ability to assert their candidacy by presenting at services, articulating their issue and articulating their ‘need’ for care.
5. Adjudication by healthcare professionals	A person’s candidacy is judged by healthcare professionals, subsequently influencing the person’s progression through services and access to care. Adjudication may disadvantage certain people by perceiving them as either ‘deserving’ or ‘undeserving’.
6. Offers of, resistance to services	A person may refuse offers at multiple stages in their journey to treatment including resisting offers for appointments, referral, and treatment.
7. Operating conditions and local production of candidacy	Incorporates factors at societal and macro levels which influence candidacy, such as the availability of local resources for addressing candidacy, and relational aspects which develop between the healthcare provider and patient over multiple visits.

Recent studies suggest the gatekeeping role of primary care in the UK, along with constrained resources [6] are contributing to an overburdened primary care [7]. The wide variety of severe and non-severe conditions being presented in primary care place pressure on GPs to adjudicate which patients need diagnostic investigations and specialist referrals. Patients themselves are also influenced by this pressure, with some reporting that they feel they should only attend primary care when their symptom is sufficiently severe to justify doing so [8].

A unique component of the Candidacy Framework is that it highlights the importance of interactions between patients and GPs, often not sufficiently addressed in similar frameworks proposed to understand access to primary care [9]. The National Institute for Health and Care Excellence (NICE) recognition and referral guidance for suspected cancer [10] emphasise the importance of the GP-patient relationship for improving early cancer diagnosis. However, research to date has mainly focused on how either patients or doctors make decisions about symptoms, without a detailed examination of the role played by interactions between patients and healthcare services.

Key processes involved in the patient journey are proposed in the Model of Pathways to Treatment [11, 12], an integrated theory outlining pathways to diagnosis from noticing symptoms to starting treatment. The model has been widely used to understand how patients make decisions about their symptoms and contacting the doctor [13–15]. Research with people experiencing cancer alarm symptoms has identified aspects of the doctor-patient relationship that influence help-seeking for possible cancer symptoms, such as the concept of ‘wasting GP time’ [16] or having previously received an ‘all-clear’ diagnosis [17].

Building on this foundation, the Candidacy Framework could be applied to more fully understand a person’s interactive journey through healthcare services. This has been shown in studies carried out in a variety of contexts [4, 5, 18], but has not yet been applied to the experience of potential cancer symptoms. We used the Candidacy Framework to focus on how doctor-patient interactions influence a person’s perceived eligibility to visit the GP.

## Methods

The present study is a secondary data analysis of interviews conducted to explore how people respond to cancer alarm symptoms. A detailed description of the study methods has been previously published [19].

### Participant selection and recruitment

Interviewees were recruited from participants in a large community survey (*n* = 2042) conducted through four General Practices in England in October, 2013. Respondents (n = 2042/4913; 42%) were asked about their experience of fourteen specific symptoms over the past 3 months, which included ten possible cancer alarm symptoms from the Cancer Awareness Measure [20], and four additional symptoms: abdominal bloating, breast changes, blood in urine and rectal bleeding. Of those participants reporting a cancer alarm symptom in the survey (*n* = 936; 46%), 602 (64%) agreed to be contacted again and were sent a follow up questionnaire. Of those responding to the follow up survey (*n* = 450; 75%), 271 (60%) reported that the symptom was still present after 3 months, and 215 (79%) consented to be contacted for interview. This formed the sampling frame for the qualitative interviews. The first 144 participants who consented to be contacted for interview were invited to participate (response rate of 60%; 86/144). Interviews were conducted by CR and KLW and ceased after data saturation was achieved (*n* = 62).

### Interviews and analysis

Interviews aimed to capture participants’ experiences of ongoing symptoms and to explore help-seeking decisions. Participants were probed about any disclosure of their symptoms to a doctor and if they had recently had a consultation in primary care, they were asked to describe their experience. Interviews lasted on average 42 min (range 22–66 min) and were digitally recorded, and transcribed verbatim. For this secondary data analysis, transcripts were analysed thematically [21] using a coding index reflecting the Candidacy Framework. Secondary data analysis is considered a valuable approach to undertake a new investigation of qualitative data [22], particularly when the primary researchers remain involved to provide contextual details [23].

**Fig. 1:**
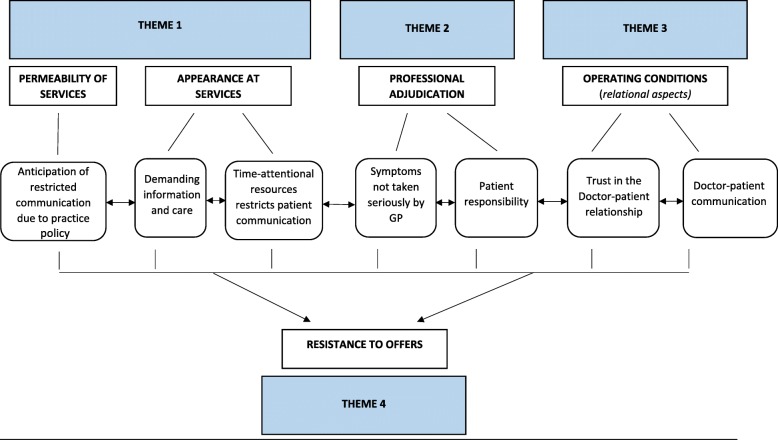
Thematic Structure set within the Candidacy Framework

ST read and re-read the transcripts to aid familiarisation and to identify relevant sections of the dialogue. Following familiarisation, each aspect of the dialogue which related to patients’ attitudes towards their GP, their experiences of healthcare, and interactions and communications within primary care were coded. Themes and sub-themes were discussed in frequent meetings between ST and KLW, and discussed with all study authors to develop the thematic structure. This structure is presented in Fig. 1 according to elements of the Candidacy Framework (Table 1). Transcripts were analysed using NVivo 9.0 software (QSR International Pty Ltd. 2010). Demographic and basic frequency information was analysed using SPSS Version 21.0.

## Results

### Sample characteristics

Sixty-two people who were experiencing cancer alarm symptoms participated in the study. The average age was 64.5 years (SD = 9.3), 53% were male, and 45% had university level education. The type of symptoms and help-seeking behaviour have been reported previously [19]. The two most common symptoms were persistent cough/hoarseness (27%) and persistent change in bladder habits (24%). Help-seeking varied by symptom: the majority of people had contacted the GP about persistent pain (7/12), but frequency of help-seeking for other symptoms was lower (e.g. 6/17 had contacted the GP about a persistent cough/hoarseness; and 2/7 about a change in mole).

### Findings

When participants talked about their use of healthcare services for potential cancer symptoms, their subjective eligibility for seeking GP help was influenced by five components of the Candidacy Framework, which provided a useful coding index for the data.

### Theme 1: Permeability of services and appearance at services

Three sub-themes emerged in relation to participants’ descriptions of the barriers to accessing services. Firstly, they described anticipating that their communication with the GP would be restricted because of various aspects of practice policy. Secondly, they described actual experiences of restrictions to communication after presenting to their GP. The third sub-theme related to difficulties asserting candidacy and demanding information and care once they had presented.

#### Anticipation of restricted communication due to practice policy

Participants expressed knowledge regarding GP surgery policies which they believed enforced time-limited communication of symptoms. These restrictions on time acted as a barrier for people when expressing the need for an appointment and selecting issues to discuss with a GP prior to their appearance at the service.

*Now, there is a standing rule in the particular surgery that I can only mention two things on each visit […] I invariably have more than two things that I could mention if time were no object, … [but] generally, there will be one significant one which is what’s provoked me to go in the first place.* (72yo; male; higher SES; sore & pain).

#### Experience of restricted communication in the doctor-patient encounter

When attending scheduled appointments, participants described how the anticipated restrictions described above were experienced in the doctor-patient encounter. One participant described how these restrictions acted as a barrier to her future help-seeking:

*You just feel like they just don’t have time to sit there and listen to what you want to say. I mean, sometimes I might go in with a piece of paper and write down my symptoms, for the doctor to push it away and not even look at it. I mean, to me, what is the point? [...] Which, to me, I thought they just don’t care, seem to bother. So yeah, I just don’t bother going unless I really have to.* (62yo, female; higher SES; pain)

#### Demanding information and care

There was a perception that patients had to be explicit and pro-active in demanding care or further information in order to receive adequate care:

*I tend not to get information given [to] me unless I ask for it.* (77yo; male; low SES; bloating, cough)

One participant described his experience of having to insist with his GP that he see a specialist in order to ‘sort out’ his concern.

*I insisted on seeing a specialist. […] But my own doctor says to me, “No, we’ll leave it alone, it looks alright. But if you find any change, let me know.” […] And like I said, if a mole starts changing colour or shape, you do something about it. And I knew that…So I said.., “No, this is a problem, I need to sort it out. And it got sorted out.”* (67yo; male; low SES; lump)

##### Theme 2: Professional adjudication

In addition to restricted communications with their GP, some participants believed their GP might not take their symptoms seriously due to biased symptom interpretation, making them feel ‘undeserving’ of care.

#### Symptoms not taken seriously

Some participants expressed concern that their GP might not take their problem seriously. For example, one woman described how previous negative adjudication from her GP acted as a barrier to seeking help via her practice, leading her to seek and receive help through an alternative route (pharmacy).

*That made me feel, well, what am I doing here, I might as well of stayed at home if they are not going to take any notice of me. And so I don’t go back because I feel like that. […] Yeah, definitely, once or twice you are laughed at as though they’ve never heard about it in their life. […] she just laughed at me. And I went to the chemist and explained the symptom, and she looked on the computer and came out with a load of paperwork which helped me find out what was wrong with me.* (62yo; female; higher SES; pain)

Participants often described feeling discouraged from seeking help for symptoms due to previous visits where their GP interpreted their concerns as age-related.

*And he just went, “Oh it’s your age.” Which they say to everything. […] And I think, forget it, I’m not even bothering, like I say, unless I’m really, really ill.* (52yo; female; higher SES; mole, change in bowel and bladder habits, persistent pain)

#### GP symptom attribution and patient responsibility

Participants also described how their GPs attributed symptoms, suggesting that symptoms were at times interpreted to be caused by age (as in the example above), obesity or smoking, sometimes implying potential patient blame and resulting in participants feeling their symptoms were dismissed without adequate investigation.

One participant described talking to a friend regarding this misattribution and dismissal of her symptoms as ‘weight-related’, and how the shame following this professional judgment led her to avoid seeking help.

*I’d even said to a friend jokingly, because we talk about things, you know, if I’m talking about something, I said, ″Honest to goodness, we could die because we don't want to go to the doctor and be told you're overweight. So you could have a symptom and […] I think I always felt everything would come back to that. And because you were slightly ashamed of that, I suppose you think you don't want that to happen.* (61yo; female; low SES; lump and unexplained pain)

Participants who reported having a cough described their apprehension in discussing this symptom with their GP, because there was an expectation that the GP would assume it was related to smoking:

*And so I suppose that’s why I felt almost now that I had myself to make that decision about… rather than go up and say, “Oh, I’ve still got this funny cough, or a little cough that I have now and then.” Do you see what I mean? It’s quite, I sort of know what someone will say [that it’s due to smoking], so I don't feel it’s anything else.* (53yo; male; low SES; persistent cough)

##### Theme 3: Operating conditions

Participants described the relationship which develops between their healthcare provider and themselves over multiple visits, which enabled the development of a trusting doctor-patient relationship, influencing help-seeking and navigation through the service.

#### Trust in the doctor-patient relationship

Having continuity of care with a single GP was described as an important aspect to enable the development of a trusting relationship. One participant described how his relationship with his GP developed over time through sympathetic interactions.

*I’ve known him for 20 years, I suppose. He knows me, and I think the continuity of treatment and the chap that knows you, and knows your history – and I know they have it all on the screens now and they all sit there doing it, and I remember him first starting that [..] But no, it’s someone you know; someone you trust; someone you think might be sympathetic.* (68yo; male; higher SES; persistent cough)

GPs were described as ‘trusted’ when they had a general understanding of patients’ health concerns, and were able to both listen to and act upon them. A participant described how these characteristics of his GP made him the first port of call for all his health concerns.

*He’s the expert I’d go to; he’s my first port of call. I would always go to him first. […] I would regard him as having the overview of all my health-related concerns. […]I trust him implicitly, actually. I’ve got a huge amount of trust with him with that overview.* (62yo; male; higher SES; difficulty swallowing)

Participants also reported negative experiences with their GPs, suggesting that they didn’t always have *“confidence that they refer you in the right way”* (65yo; female; higher SES; change in bowel habits).

#### Doctor-patient communication as the foundation for the relationship

The role of communication was described as a key component of the doctor-patient relationship. One participant described how miscommunication can occur when the GP lacks empathetic communication and is unable to adequately acknowledge the patient’s worry.

*I don’t know, they might just joke with you, and things like that. And you feel like, sorry, but I feel like crying, not laughing. And they’ll perhaps talk about something else when you are trying to get across how you feel.* (62yo; female; higher SES; pain)

Participants described challenging interactions with GPs where their condition was not fully explained to them, or where information given was not clearly understood. This left some participants feeling confused and unsupported:

*Half the time, if you ask for information, you get something and you don’t understand it anyway. Which then leads me to go home and look it up, and then I’m terrified.[…]* (77yo; male; low SES; bloating, cough)

##### Theme 4: Resistance to offers

Participants described declining available appointments if they were with an unfamiliar doctor with whom they did not have a trusting relationship, and resisting returning to services with repeated or persistent symptoms. Some participants described a preference for delaying an appointment to wait for the availability of a preferred or trusted doctor with whom they felt comfortable discussing their symptoms:

*“Yes. If they say, “Dr So-and-So will see you,” I’ll say, “Well no, I’ll leave it another day, please. Can I see someone else?” Because it seems to me pointless to go and see the doctor if you are not going to feel comfortable or reassured with the session you are having with them”.* (77yo; female; low SES; bladder habits and bloating)

Another participant described the importance of having confidence in his GP to know ahead of time that all his concerns would be heard, despite the time-restrictions of his GP appointment:

*“If I go with two or three things, I know he will listen to it. Even when I know and he knows he’s only got ten minutes […] I’m quite confident that he wouldn’t just whip through the second one as though that’s not what you’ve come for. He will reassure me about it”.* (62yo; male; higher SES; unexplained pain/abdominal bloating)

On the other hand, another participant described how despite the persistent discomfort caused by his symptoms, he did not believe that the GP was capable of resolving his existing concern, given that his issue was not resolved during his first visit to the GP.

“*Yeah. It just depends on the time. Sometimes it can wear me down a bit. If it’s there for a few days, I get a bit weary with it and then I’ll, sort of, think, oh I’ll go back to the doctor’s. But I think, well, he’s done everything he can”.* (62yo; male; higher SES; unexplained pain/abdominal bloating)

## Discussion

We found that several components of the doctor-patient relationship influenced perceived eligibility to seek medical help in the context of possible cancer symptoms. These components mapped onto five stages of the Candidacy Framework [1], including permeability of services (ease of using health services), appearance at services (articulating need), adjudication by doctors (perception of patient as deserving or undeserving), and resistance to offers (e.g. patient resisting appointments and/or referrals).

Previous studies have identified difficulties in access to care, focusing primarily on organisational or system level processes that act as barriers to utilisation of primary care services [9], or person-centred barriers using the Model of Pathways to Treatment [8]. Other studies have evaluated the effect of doctor-patient communication on diagnostic delay [24], and emphasised the importance of doctors’ decision making [25]. Using the Candidacy Framework allowed us to go further and explore the GP-patient relationship taking the different components into account, from service level factors to individual level characteristics of the healthcare professional and the patient.

Our findings also resonate with other theoretical frameworks, such as Social Cognitive Theory (SCT: [26]), where self-efficacy (belief in the ability to succeed) and outcome expectations (perceived consequences of action) influence whether someone will seek medical help. For example, a person’s ability to assert candidacy and articulate their issue requires self-efficacy, as well as the belief that seeking medical help will result in a useful outcome (e.g. referral to a specialist). SCT also takes into account socio-structural barriers/ opportunities such as having a pre-booked appointment [11]. However, the Candidacy Framework provides more detailed specification of these factors in the healthcare context and highlights the continual negotiation between patients and health services. For example, the Candidacy Framework stipulates the importance of relational aspects between the doctor and patient and how these develop over multiple visits (Table 1).

The allocation of resources and system policies in a general practice, including limited appointment availability, fixed appointment length [8] and time restriction within consultations [27], influenced the patient’s ability to articulate their problems and their perceived entitlement to care when anticipating booking appointments and appearing at services.

Adjudication from the GP has previously been shown to influence candidacy and receipt of care [1, 4, 28], and our findings suggest that this encounter may be influenced by these policy and system pressures which prioritise certain patient concerns and may encourage judgment of patients as ‘deserving’ or ‘undeserving’ of care, even in the context of experiencing well known cancer ‘alarm’ symptoms such as a change in a mole.

Deciding on whether symptoms are eligible for help-seeking is challenging, particularly for patients with vague or intermittent symptoms [15], or chronic health conditions, where worry about wasting the doctor’s time is a commonly reported patient barrier [3, 16]. Findings suggest that adjudication of symptom severity may lead patients to feel that their symptoms are not being taken seriously by their GP [29], or are misattributed to comorbid conditions such as obesity or smoking-related conditions, resulting in missed opportunities for diagnosis [27].

The GP-patient relationship, formed during encounters with the primary care service, can act both as a facilitator of candidacy or a barrier to care, particularly when considering accepting an offer to meet with a different GP, or neglecting to return to services with persistent symptoms. Results support previous findings which suggest GP safety-netting procedures may not sufficiently engage patients to return to services if symptoms do not resolve [30], and that needing to visit the GP multiple times to address their concerns may lead to reduced satisfaction with services and impact the GP-patient relationship [31]. A lack of confidence in the GP’s abilities to help may also influence help-seeking for alarm-symptoms, as a result of incorrect adjudication, or over-reassurance and under-support following a previous false-alarm [17]. Our findings thus demonstrate that these influences have implications for both current and future consultations.

To our knowledge, this is the first study to apply the Candidacy Framework to understand how aspects of the doctor-patient relationship influence perceived eligibility and help-seeking for people experiencing cancer alarm symptoms. Future research could focus on specific population sub-groups, potentially including those with comorbid conditions to explore how this influences help-seeking and reporting of cancer alarm symptoms. Interviewing patients at one time point does not allow for an exploration of the complete patient journey, or provide the GP perspective. Given the secondary nature of the analysis, in which the Candidacy Framework was applied to existing interview data, future research should explore the doctor-patient conversation in relation to cancer alarm symptoms more fully (e.g. by including the healthcare provider perspective), and ideally without the potential bias associated with conducting retrospective interviews. Although the study was conducted in a UK context, the findings may be applicable to countries with similar healthcare systems.

## Conclusions

The majority of cancers are diagnosed following presentation in primary care. Given the drive to improve earlier diagnosis of cancer, this study used the Candidacy Framework to explore how the doctor-patient relationship influences perceived eligibility for accessing primary care. Several dimensions emerged, including commonly reported issues such as the sometimes impermeable nature of healthcare services, as well as less explored issues regarding how doctors’ symptom appraisal, cognitive biases, and communication strategies influence the conversation in primary care. Understanding how the doctor-patient relationship may impact on help-seeking for cancer symptoms could inform the development of effective strategies to empower both patients and GPs, resulting in improved primary care encounters and ultimately earlier diagnosis of cancer.

For example, permeability of services may be improved by addressing the issue of perceived time restrictions for appointments, by allowing patients to speak with primary care nurses prior to seeing their GP. However, our findings suggest that this is only one of many practical components that influence candidacy. Interventions may also need to consider training to support GP-patient communication to encourage candidacy among patients. Training both patients and GPs in a ‘push-pull’ approach has shown promise. For example, one study reported encouraging evidence that a public awareness intervention, paired with a brief training intervention in general practice, increased symptom reporting and referrals for lung cancer [32].

Good listening skills of the doctor are not only the most important attribute when patients consider different consultation options for possible cancer symptoms [33], but good communication at the GP practice level is also associated with being more likely to investigate and refer patients in the cancer context [34]. Given that healthcare professionals play a key role not only in early detection and diagnosis, but also in facilitating future help-seeking and healthcare utilisation, this framework highlights areas for targeted interventions to improve patient-centred care and encourage help-seeking behaviours. Many existing interventions designed to improve access to healthcare focus on health services at the system level, or public health awareness campaigns. In order to translate health awareness into patients’ perceived eligibility for healthcare and ability to articulate needs and access services, it is essential that interventions also take into account the complex interconnections of health, cognitive factors and social structural dimensions of care within the GP-patient relationship [9].
